# Transaminase Activity Predicts Survival in Patients with Head and Neck Cancer

**DOI:** 10.1371/journal.pone.0164057

**Published:** 2016-10-12

**Authors:** Yukinori Takenaka, Norihiko Takemoto, Toshimichi Yasui, Yoshifumi Yamamoto, Atsuhiko Uno, Haruka Miyabe, Naoki Ashida, Kotaro Shimizu, Susumu Nakahara, Atshushi Hanamoto, Takahito Fukusumi, Takahiro Michiba, Hironori Cho, Masashi Yamamoto, Hidenori Inohara

**Affiliations:** 1 Department of Otorhinolaryngology-Htaead and Neck Surgery, Osaka General Medical Center, Sumiyoshi, Osaka, Japan; 2 Department of Otorhinolaryngology-Head and Neck Surgery, Osaka University Graduate School of Medicine, Suita, Osaka, Japan; University of Cincinnati College of Medicine, UNITED STATES

## Abstract

Various serum biomarkers have been developed for predicting head and neck squamous cell carcinoma (HNSCC) prognosis. However, none of them have been proven to be clinically significant. A recent study reported that the ratio of aspartate aminotransaminase (AST) to alanine aminotransaminase (ALT) had a prognostic effect on non-metastatic cancers. This study aimed to examine the effect of the AST/ALT ratio on the survival of patients with HNSCC. Clinical data of 356 patients with locoregionally advanced HNSCC were collected. The effect of the AST/ALT ratio on overall survival was analyzed using a Cox proportional hazard model. Moreover, recursive partitioning analysis (RPA) was used to divide the patients into groups on the basis of the clinical stage and AST/ALT ratio. The prognostic ability of this grouping was validated using an independent data set (N = 167). The AST/ALT ratio ranged from 0.42 to 4.30 (median, 1.42) and was a prognostic factor for overall survival that was independent of age, primary sites, and tumor stage (hazard ratio: 1.36, confidence interval: 1.08−1.68, P = 0.010). RPA divided patients with stage IVA into the following two subgroups: high AST/ALT (≥2.3) and low AST/ALT (<2.3) subgroups. The 5-year survival rate for patients with stage III, stage IVA with a low AST/ALT ratio, stage IVA with a high AST/ALT ratio, and stage IVB were 64.8%, 49.2%, 28.6%, and 33.3%, respectively (p < 0.001). Compared with the low AST/ALT group, the adjusted hazard ratio for death was 2.17 for high AST/ALT group (confidence interval: 1.02–.22 P = 0.045). The AST/ALT ratio was demonstrated to be a prognostic factor of HNSCC. The ratio subdivided patients with stage IVA into low- and high-risk groups. Moreover, intensified treatment for the high-risk group may be considered.

## Introduction

Cancer prognosis is predicted with respect to the primary tumor size, extent of tumor invasion, spread to lymph nodes and distant organs, and levels of biomarkers. Various biomarkers have been developed to predict head and neck squamous cell carcinoma (HNSCC) prognosis [1]. Using tumor tissue specimens or blood samples, these biomarkers are measured. However, tissue biomarkers that are measured using a biopsy specimen do not always reflect the nature of the whole tumor. Furthermore, tissue biomarker studies frequently demonstrate contradictory results because of variations in tissue processing (fresh or fixed) and scoring procedures [1]. Obtaining specimens through invasive procedures sometimes limit the availability. On the other hand, blood biomarkers are easily obtained through non-invasiveness procedures. Leukocyte count, serum inflammatory marker levels, and serum SCC antigen levels are reliable prognostic markers for HNSCC [2–4]. However, these prognostic blood biomarkers rarely provide additional prognostic information regarding the TNM staging. Therefore, despite having prognostic abilities, these biomarkers are not clinically useful.

Alanine aminotransaminase (ALT) and aspartate aminotransaminase (AST) are routinely measured before treating HNSCC. The AST/ALT ratio (De Ritis ratio) has been used to differentiate liver diseases [5, 6]. Bezan et al. [7] recently demonstrated the AST/ALT ratio was a prognostic factor for non-metastatic renal cell carcinoma, indicating that the AST/ALT ratio may have a prognostic effect in patients with cancers who did not have a liver disease. These results prompted us to investigate the prognostic role of the AST/ALT ratio in patients with HNSCC.

This study aims to elucidate the prognostic effect of serum transaminase activity in patients with HNSCC and to investigate whether the AST/ALT ratio provides additional prognostic information to the TNM staging.

## Materials and Methods

### Ethics Statement

The protocol of the present study was approved by the Institutional Review Board of Osaka University and Osaka General Medical Center. All patients provided written informed consent.

### Patients and data extraction

#### Training set

The medical charts of all patients with previously untreated locoregionally advanced SCC of the oropharynx, hypopharynx, larynx, and oral cavity who were treated in the Osaka University Hospital between January 2004 and December 2012 were subjected to a retrospective review. Our cancer registry system identified 391 consecutive patients with histologically confirmed HNSCC. Thirty-five patients were excluded because the period of observation was less than 24 months and their clinical or laboratory data were insufficient. Finally, 356 patients were included in the training set. The clinicopathological characteristic and laboratory results of the resultant 356 patients were used to assess the association of transaminase activity, AST/ALT ratio, and prognosis and to determine the cut-off value of the AST/ALT ratio. The clinical stage was determined according to the Union for International Cancer Control TNM classification system, 7th edition. The initial workup for these patients included a contrast-enhanced computed tomography (CT) of the neck, 18F-fluorodeoxyglucose positron emission tomography (FDG-PET) with or without CT, and panendoscopy.

#### Validation set

We employed an independent dataset for validating the AST/ALT ratio as a prognostic factor in patients with HNSCC. In particular, we searched the cancer registry of the Osaka General Medical Center to identify patients with locoregionally advanced HNSCC who were treated between 2006 and 2012. We identified 189 consecutive patients with histologically confirmed SCC of the oropharynx, hypopharynx, larynx, and oral cavity. Among them, 22 patients were excluded because of a follow-up period of <24 months and insufficient clinical or laboratory data. The final number of patients for analysis was 167. The data of these patients were used to estimate survival rates and hazard ratios (HRs) according to the risk groups.

### Measurement of serum transaminase activity

AST and ALT levels were measured at the first visit using the consensus method of the Japan Society of Clinical Chemistry (JSCC) for serum AST and ALT [8]. The JSCC consensus method is closely correlated with the method of the International Federation of Clinical Chemistry (IFCC). However, the JSCC method tends to reveal lower AST and ALT levels compared with the IFCC method because pyridoxal phosphate is not used.

### Statistical analysis

The AST/ALT ratio was analyzed as a continuous or ordinal variable. The chi-square test was used to assess the associations between categorical variables, and the Mann—Whitney or Kruskal—Wallis test was used to assess the associations between categorical and continuous variables. Survival was estimated using the Kaplan—Meier method and was compared using the log-rank test. A Cox proportional hazard model was used to obtain HR for death. A *p* value of <0.05 was considered to be statistically significant. Recursive partitioning analysis (RPA) was used to divide the patients into risk-based groups according to the clinical stage and AST/ALT ratio. The JMP v12 statistical software (IBM Japan, Tokyo, Japan) was used to perform statistical analyses.

## Results

### Characteristics of patients in the training set

The clinicopathological characteristics of patients are summarized in Table 1. The male-to-female ratio was 7:1, and the median age was 66 (range, 42–92) years. Forty-five percent of patients were in their seventh decade. The most common primary tumor site was the hypopharynx (38%), followed by the oropharynx (31%), larynx (18%), and oral cavity (13%). The clinical stage of these tumors was III (32%), IVA (60%), and IVB (8%). Chemoradiation therapy (CRT) was performed in 75% of patients. The median value and range of AST levels, ALT levels, and the AST/ALT ratio were 22 (5–143) U/L, 15 (4–184) U/L, and 1.42 (0.42–4.30), respectively. The number of patients with AST levels above the normal range (AST > 32 U/L) and those with ALT levels above the normal range (ALT > 37 U/L) were 64 (18%) and 36 (10%), respectively. The median follow-up period for the surviving patients was observed to be 57.2 months (range, 24.6–125.3).

**Table 1:** Patient characteristics by primary sites.

	training set		validation set		
	No. (n = 356)	%	No. (n = 167)	%	*P* value
Sex					
male	312	87.6	117	70.1	<0.001
female	44	12.4	50	29.9	
Age, years					
median, range	66, 42–92		67, 28–90		0.005
Primary site					
hypopharynx	136	38.2	31	18.6	<0.001
oropharynx	110	30.9	27	16.2	
larynx	64	18	34	20.4	
oral cavity	46	12.9	75	44.9	
T classification					
1/2/3/4	30/105/112/119	8.4/29.5/31.5/30.6	12/42/36/77	7.2/25.2/21.6/46.1	0.006
N classification					
0/1/2/3	69/75/197/15	19.4/21.1/55.3/4.2	39/42/80/6	23.4/25.2/47.9/3.6	0.391
Stage					
III/IVA/IVB	113/213/30	31.7/59.8/8.4	47/111/9	28.1/66.5/5.4	0.249
Treatment modality					
surgery	63	17.7	90	53.9	<0.001
* (C) RT	268	75.3	55	32.9	
other	25	7	22	13.1	
AST					
median, range	22, 5–143		23, 10–166		0.034
ALT					
median, range	15, 4–184		17, 4–79		0.324
AST/ALT ration					
median, range	1.42, 0.42–4.30		1.46, 0.54–4.88		0.300

* (C) RT, (chemo-) radiation therapy

### AST/ALT ratio as a prognostic factor

To investigate whether AST levels, ALT levels, and the AST/ALT ratio have a prognostic effect, a Cox proportional hazard model was used (Table 2).

**Table 2:** Overall survival according to clinicopathologic variables and AST/ALT ratio.

	Univariate analysis			Multivariate analysis		
clinicopathologic variable	hazard ratio	95% confidence interval	*P* value	hazard ratio	95% confidence interval	*P* value
Sex						
Male / Female	1.01	0.66–1.62	0.966			
Age (y)						
≥65 / <65	1.51	1.12–2.05	0.007	1.65	1.21–2.25	0.001
Primary site						
hypopharynx	2.01	1.29–3.25	0.002	1.76	1.10–2.92	0.019
oropharynx	1.08	0.66–1.83	0.758	1.02	0.61–1.75	0.954
larynx	Ref			Ref		
oral cavity	1,87	1.06–3.32	0.031	2.08	1.17–3.71	0.013
Stage						
III	Ref			Ref		
IVA	1.42	1.01–2.03	0.045	1.39	0.97–2.02	0.074
IVB	2.07	1.19–3.46	0.011	2.07	1.15–3.60	0.016
AST(continuous)						
	1.00	1.00–1.01	0.344			
ALT(continuous)						
	0.99	0.98–1.00	0.240			
AST / ALT ratio (ordinal)						
1st quartile (<1.09)	Ref					
2nd quartile (≥1.09, <1.42)	1.72	1.09–2.77	0.020			
3rd quartile (≥1.42, <1.82)	1.90	1.21–3.04	0.005			
4th quartile (≥1.82)	2.19	1.40–3.51	0.001			
AST / ALT ratio (continuous)						
	1.44	1.15–1.76	0.002	1.36	1.08–1.68	0.010

Univariate analysis of overall survival demonstrated that age, primary site, and stage were the significant prognostic factors for overall survival. Neither AST nor ALT was a significant factor. When the AST/ALT ratio was analyzed as an ordinal variable, HR for second, third, and fourth quartile compared to the first quartile were 1.72, 1.90, and 2.19, respectively (95%CI: 1.09–2.77, 1.21–3.04, and 1.40–3.51, p = 0.020, 0.005, and 0.001, respectively). This indicates that the AST/ALT ratio and HR for death have a linear relationship. Similarly, the AST/ALT ratio as a continuous variable was a significant prognostic factor (HR 1.44, 95%CI: 1.15–1.76, p = 0.022). To exclude possible confounding factors, a multivariate analysis was performed, and the results revealed that the AST/ALT ratio (HR 1.36, 95%CI: 1.08–1.68, p = 0.010), older age, hypopharynx primary, oral cavity primary, and stage IVB were all independent prognostic factors for overall survival.

### The AST/ALT ratio and TNM staging

The associations between the AST/ALT ratio and T classification, N classification, and stage are shown in Table 3.

**Table 3:** Association of DeRitis ratio and TNM staging.

	De Ritis ratio		
	median	range	p value
T			0.435
T1	1.3	0.75–3.00	
T2	1.32	0.45–4.30	
T3	1.49	0.44–4.00	
T4	1.47	0.42–3.50	
N			0.180
N0	1.39	0.42–3.33	
N1	1.44	0.68–3.28	
N2	1.39	0.44–4.30	
N3	1.83	0.76–2.75	
Stage			0.369
III	1.44	0.48–3.29	
IVA	1.38	0.42–4.30	
IVB	1.64	0.59–2.75	

Although the median value of the AST/ALT ratio was high in advanced T classification, the difference was insignificant (p = 0.435). Similarly, there were no significant associations between N classification and the AST/ALT ratio (p = 0.180) and between stage and the AST/ALT ratio (p = 0.369).

### Classification of patients according to the stage and AST/ALT ratio

**Fig 1:**
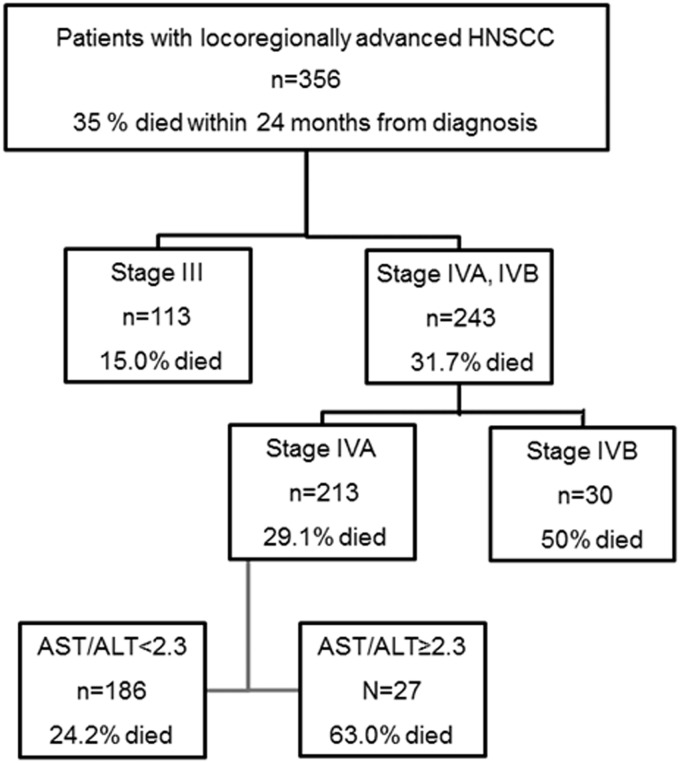
Recursive partitioning analysis was used to classify patients into different risk groups according to clinical stage and AST/ALT ratio.

RPA was performed to identify patients at a high risk. As shown in Fig 1, The AST/ALT ratio subdivided patients with stage IVA into either a favorable or poor prognosis group.

The cut-off value of the AST/ALT ratio was 2.3. The grouping demonstrated here was employed for further analyzes.

### External validation

**Fig 2:**
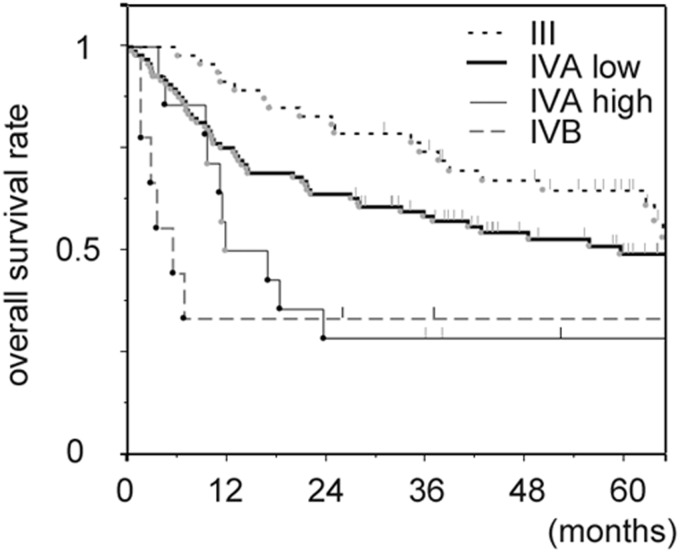
Kaplan-Meier curves for overall survival according to the groups defined by the recursive partitioning analysis.

An independent dataset was used to validate the prognostic effect of the AST/ALT ratio. Characteristics of patients between the training and validation sets were significantly different in the distribution of primary sites, T classification, treatment modality, and AST level (Table 1). The validation set included more number of female patients, patients with T4 disease, and patients with oral cavity primary. In the training set, the most employed treatment was CRT, while in the validation set, it was surgery. In the validation set, the median follow-up period for surviving patients was 59.9 (range, 25.9–111.4) months. According to the classification that was determined using the training set, the distribution of each group in the validation set was as follows: stage III, 47 patients (28%); stage IVA with low AST/ALT, 97 patients (58%); stage IVA with high AST/ALT, 14 patients (8%); and stage IVB, 9 patients (5%). The overall survival curve according to this classification is shown in Fig 2.

The 5-year survival rate for patients with stage III, stage IVA with low AST/ALT, stage IVA with high AST/ALT, and stage IVB were 64.8%, 49.2%, 28.6%, and 33.3%, respectively (p < 0.001). Compared with stage IVA with low AST/ALT group, HRs (adjusted for age, site and treatment modality) were 0.71 (95%CI: 0.40–1.20 p = 0.204), 2.17 (95%CI: 1.02–4.22 p = 0.045), and 2.64 (95%CI: 1.06–5.66 p = 0.038) for stage III, stage IVA with high AST/ALT, and stage IVB groups, respectively (Table 4).

**Table 4:** Adjusted hazard ratio according to stage and AST/ALT ratio.

	hazard ratio*	95% confidence interval	*P* value
Stage III	Ref		
Stage IVA, AST/ALT<2.3	1.41	0.83–2.49	0.204
Stage IVA, AST/ALT≥2.3	3.07	1.33–6.68	0.001
Stage IVB	3.74	1.41–8.85	0.001

*Hazard ratio was adjusted for age, site and treatment modality.

## Discussion

Predicting the prognosis of patients with HNSCC using blood samples has been conducted for many years [1, 9]. Blood biomarkers, such as neutrophil count, monocyte count, platelet count, serum C-reactive protein level, serum albumin level, and interleukin-6 level, for the systemic inflammatory response are associated with cancer prognosis [4, 9–12]. Among the markers and scoring systems based on the inflammatory response, the Glasgow prognostic score, which is determined with serum albumin and C-reactive protein levels, is the most prevalent because of easy measurement and simplicity [13, 14]. The Glasgow prognostic score has been validated as a reliable prognostic factor of HNSCC [2]; however, its clinical utility is limited because it neither has better prognostic ability than the TNM staging nor provides additional prognostic information to the TNM staging. Furthermore, the serum tumor-specific markers, such as serum SCC antigen, have prognostic values but have similar problems as the Glasgow prognostic score [3]. Therefore, no blood biomarkers for HNSCC affect treatment decision and have clinical significance. On the other hand, saliva and serum biomarkers for diagnostic purpose have been proven to be useful to some extent. [15] [16]

The De Ritis ratio is the proportion of serum AST to ALT levels, which was described by De Ritis et al. in 1957 [5]. This ratio is used for diagnosing and clinical decision-making of liver diseases [6]. Recent studies by Bezan et al. [7] demonstrated that the AST/ALT ratio is a prognostic factor in non-metastatic renal cell carcinoma, independent of T and N classification. Moreover, they reported that incorporating the AST/ALT ratio to the previously proven prognostic score improved the prognostic value. However, to date, there have been no reports regarding the prognostic value of the AST/ALT ratio in patients with other types of cancer, including HNSCC.

This study focused on the role of the AST/ALT ratio in patients with HNSCC. We found that the AST/ALT ratio had no significant associations with T and N classifications and was an independent prognostic factor for overall survival. In addition, we validated the prognostic effect of the AST/ALT ratio with the independent data set. The AST/ALT ratio further divided patients with stage IVA into poorer and better prognosis subsets.

The association of cancer and transaminases has been mainly discussed regarding liver metastasis or liver invasion. In patients with breast cancer having liver metastasis, an elevated AST level was an independent prognostic factor [17]. AST level was associated with poor prognosis in patients with advanced pancreatic cancer [18]. However, only a few studies have investigated the role of transaminase in malignant disease except for liver cancer and their results were inconsistent [19, 20]. Chougule et al. [19] reported that AST and ALT levels in patients with stage II and III head and neck cancer and those with cervical cancer were much higher than those in healthy individuals. Moreover, with continuous radiation therapy, the elevated transaminase levels normalized. Conversely, Proctor et al. [20] demonstrated that patients with cancer, in general, had lower AST and ALT levels. However, in our study, many patients demonstrated normal AST and ALT levels, while a small proportion of patients had high AST and ALT levels. While this study used the JSCC method to measure transaminase levels, previous studies did not describe the measurement method used. The types of cancer and their clinical stage varied among each study. The differences in measurement methods and patients’ characteristics may explain, at least in part, the discordance.

Many key oncogenic signaling pathways converge to adapt to tumor cell metabolism [21]. Hence, alterations in cellular metabolism should be considered a crucial hallmark of cancer. Cancer cells acquire alterations during carbohydrate, protein, lipid, and nucleic acid metabolism. The best characterized metabolic phenotype that was observed in tumor cells is the Warburg effect. Warburg demonstrated that cancer cells metabolized more glucose than normal cells, with enhanced aerobic glycolysis and pyruvate production [22, 23]. Furthermore, glutaminolysis is enhanced in cancer cells to supplement tricarboxylic acid cycle metabolites [23]. ALT, which catalyzes the conversion of pyruvate and glutamate to alanine and α-ketoglutarate [6], functions in both glycolysis and glutaminolysis. Therefore, compared with serum AST levels, serum ALT level decreases (increased AST/ALT ratio) could be a manifestation of enhanced metabolism and consumed ALT in aggressive cancer cells; Conde et al. [24] supported this speculation. *In vitro* experiments with a bladder cancer cell line revealed decreased ALT levels in more invasive cells compared with less invasive cells. Furthermore, more invasive bladder cancer cells consumed glucose and pyruvate to a greater extent.

FDG-PET enables the clinical detection of glucose metabolism alteration in cancer and is used to predict initial staging, follow-up, clinical outcome, and second primary cancer screening of HNSCC [25, 26]. In addition, volumetric PET/CT parameters can predict a response to chemoradiation therapy in patients with HNSCC [27]. Therefore, FDG-PET parameters can be used as predictive and prognostic markers. Moreover, PET of glutaminolysis in tumors has been studied to compensate for the shortcomings of FDG-PET [28]. Thus, detecting cancer cell metabolism with nuclear imaging has been integrated into routine clinical application, and its application continuous to improve. In our study, the patients with stage I and II demonstrated the AST/ALT ratio was so low that analysis results were not significant (data not shown). This might reflect that above a certain level of the tumor volume with hypermetabolism is needed to cause a difference in the AST/ALT ratio. From the results of this study, we inferred that measuring of the AST/ALT ratio could substitute metabolic imaging in both its prognostic and predictive roles.

There are several limitations in this study. First, it was a retrospective study. We could not obtain sufficient information regarding 9.2% and 11.6% of patients in the training and validation sets, respectively, thereby resulting in some bias in the results. Second, the measurement methods for serum AST and ALT in Japan are different from those in other countries. Both the JSCC and IFCC methods should be used and compared in future studies. Third, HPV status should be considered for analyzing oropharyngeal cancer. Finally, the mechanisms of how the AST/ALT ratio is associated with survival of patients with HNSCC remain to be studied.

In conclusion, we demonstrated that the AST/ALT ratio is a prognostic factor of HNSCC. Among patients with stage IVA HNSCC, the subset with a high AST/ALT ratio had the worse prognosis. Therefore, they can be considered as candidates for intensified treatment. Moreover, the metabolic change that was observed as an increased AST/ALT ratio might be a substitute for a prognostic role of FDG-PET. Because of easy measurement and its potential utilities, further studies on this topic should be encouraged.

## Supporting Information

S1 Table Patient characteristics in training set. (XLSX)

S2 Table Patient characteristics in validation set. (XLSX)
